# Mitochondrial DNA Damage and Histological Features in Liver Tissue of Azoxymethane-Treated *Apex1* Haploinsufficient Mice

**DOI:** 10.3390/biom15121706

**Published:** 2025-12-06

**Authors:** Carmen M. Pérez-Pérez, Adlin Rodríguez-Muñoz, Gerardo G. Mackenzie, Karen E. Matsukuma, María R. Castro-Achi, Sylvette Ayala-Peña, Carlos A. Torres-Ramos

**Affiliations:** 1 Department of Physiology, University of Puerto Rico Medical Sciences Campus, San Juan, PR 00936, USA; 2 Department of Nutrition, University of California Davis, Davis, CA 95616, USA; 3 Department of Pathology and Laboratory Medicine, University of California Davis, Davis, CA 95616, USA; 4 Department of Pharmacology and Toxicology, University of Puerto Rico Medical Sciences Campus, San Juan, PR 00936, USA

**Keywords:** mitochondria, base excision repair, liver

## Abstract

Mitochondrial dysfunction and loss of mitochondrial DNA (mtDNA) integrity are increasingly recognized as key contributors to liver diseases such as cirrhosis and hepatocellular carcinoma. However, the role of mtDNA repair in maintaining mitochondrial homeostasis during liver injury remains poorly understood. Apurinic/apyrimidinic endonuclease 1 (APE1), encoded by the *Apex1* gene, is the primary endonuclease mediating base excision repair of mtDNA. We hypothesize that APE1 is required to preserve mtDNA integrity in response to genotoxic stress in the liver. To test this, wild-type (WT) and *Apex1* haploinsufficient mice (*Apex1^+/−^*) were treated with the alkylating agent azoxymethane (AOM), a carcinogen bioactivated in the liver, and tissues were collected 20 weeks after the last exposure. *Apex1^+/−^* mice exhibited a 3.2-fold increase in mtDNA lesions and a 55% reduction in mtDNA abundance, changes not observed in WT mice. Bioenergetics profiling revealed a 1.5-fold increase in the ATP5β/GAPDH ratio in WT mice and a 2.5-fold increase in *Apex1^+/−^* mice, indicating a more pronounced shift toward oxidative phosphorylation in the absence of full APE1 function. Histological analysis indicated increased nuclear inclusions and ductular proliferation in both strains, whereas fibrosis was attenuated in *Apex1^+/−^* mice. Collectively, these findings show that APE1 is essential for preserving mtDNA integrity and regulating bioenergetics and histopathological responses to alkylation-induced liver injury, highlighting its dual role in mitochondrial maintenance and modulating inflammatory outcomes.

## 1. Introduction

The pathogenesis of liver diseases is a multifactorial process influenced by both environmental and genetic factors, ultimately impairing hepatic function [1]. Chronic liver disease and cirrhosis are among the strongest risk factors for hepatocellular carcinoma (HCC) [2]. However, the metabolic alterations that accompany chronic liver disease and their contribution to liver carcinogenesis remain incompletely understood. Strong evidence has highlighted the role of mitochondrial DNA (mtDNA) damage and mutations in disrupting mitochondrial function, altering energy metabolism, increasing reactive oxygen species (ROS) production, and modulating apoptosis [3,4,5,6,7]. Multiple types of mtDNA alterations, such as point mutations (both in the D-loop and coding regions), deletions, insertions, and copy number changes, have been identified in human cancers, including HCC [8,9,10]. Together, these alterations can compromise mitochondrial integrity and function, thereby promoting disease progression. One proposed mechanism linking mtDNA defects to HCC is the increased generation of mitochondrial ROS, which may drive both cancer initiation and progression [11].

Mutations in mtDNA can arise from exposure to carcinogens, oxidative stress, aging, and other insults. Azoxymethane (AOM) is a well-established carcinogen widely used to induce colorectal cancer (CRC) in rodent models [12,13,14,15]. AOM is converted in the liver to its active metabolite, methylazoxymethanol (MAM), which undergoes further activation to generate highly reactive alkylating species. These metabolites form DNA adducts, predominantly at the N7 position of guanine and the N3 position of adenine [16]. The resulting DNA lesions are primarily repaired by base excision repair (BER), a pathway critical for correcting small base modifications, such as those induced by AOM [17]. Beyond its use in CRC models, AOM has also been employed to investigate liver carcinogenesis, including studies evaluating the combined effects of AOM and high-fat diets. The liver pathology observed in these models has provided important insights into how dietary and chemical exposures interact to drive liver injury and tumor development [18,19].

Apurinic/apyrimidinic endonuclease 1 (APE1), encoded by the *Apex1* gene, is a central component of BER due to its multifunctional enzymatic activities [20]. These encompass endonuclease activity targeted at abasic sites, 3’ phosphodiesterase function, and 3’ to 5’ exonuclease, proofreading 3’ mismatches during BER, incision at distinct base lesions within the DNA duplex, and incision at abasic sites within RNA substrates. Additionally, APE1 has a redox regulatory function, which modulates the activity of transcriptional factors such as AP-1, p53, and NF-κB, thereby influencing cellular responses to oxidative stress [21]. The mechanism of APE1’s redox activity is not fully understood, as only Cys65 has been identified as critical for redox function, primarily using in vitro assays [22]. However, recent evidence indicates a role for Cys65 in in vivo redox signaling [23]. Because of the dual role in DNA repair and redox signaling, APE1 has emerged as a promising target for therapeutic interventions in a variety of human diseases, including inflammatory bowel disease and various cancer types [22,23,24,25].

Beyond its nuclear functions, APE1 also plays essential roles in mitochondria, where it safeguards mtDNA integrity [26,27]. Our previous work demonstrated that APE1 is required to maintain mtDNA integrity in colorectal tissue following both short- and long-term exposure to AOM [28]. Since AOM is bioactivated in the liver, this organ represents a primary target of its genotoxic effects and a relevant model of liver injury [18,19]. Based on these observations, we hypothesize that APE1 is required to preserve mtDNA integrity and regulate mitochondrial and histopathological responses to alkylation-induced liver injury. To test this, we examined the effects of AOM in wild-type and *Apex1* haploinsufficient mice, focusing on mtDNA damage, bioenergetics status, and liver pathology.

## 2. Materials and Methods

### 2.1. Animals and Azoxymethane (AOM) Treatment

Liver tissues were obtained from male wild-type (WT) and *Apex1* heterozygous (*Apex1^+/−^*) mice in the C57BL/6J background [29]. The initial breeding pair for our colony was generously provided by Dr. Christi Walter from the University of Texas Health Science Center at San Antonio. Genotyping was conducted using DNA isolated from tail tissue samples from each mouse collected at 21 days of age. PCR was carried out using primers designed to detect a Neomycin resistance transgene, which is only present in the *Apex1* knockout mice, as described previously [29,30]. Mice (6-month-old) were administered 10 mg/kg AOM once per week for a duration of 4 weeks. Six months after the first AOM injection, the animals were euthanized by cervical dislocation by trained personnel according to American Veterinary Medical Association (AVMA) Guidelines for the Euthanasia of Animals. Liver tissue was then removed, frozen, or fixed in 10% formaldehyde, embedded in paraffin, and stored for subsequent analysis. Mice were housed in two per cage with a 12 h light-dark cycle and ad libitum access to food and water. All animal procedures and experiments were conducted in accordance with international guidelines for the care and handling of experimental animals. The Institutional Animal Care and Use Committee and the Institutional Biosafety Committee at the University of Puerto Rico Medical Sciences approved the research protocols.

### 2.2. DNA Isolation and Quantification from Frozen Liver Tissue

DNA from liver samples was isolated using the DNeasy Blood & Tissue Kit (Quiagen, Germanton, MD, USA) as previously published [28]. The integrity of the genomic DNA was verified by running a 1% ethidium bromide (EtBr) stained agarose gel and visualizing it under UV light. Molecular weight was determined using a Lambda DNA Hind III digest (Sigma-Aldrich, Inc., St. Louis, MO, USA). DNA was quantified using the PicoGreen dsDNA Quantitation Kit (Fisher Scientific, Waltham, MA, USA), which employs a fluorescent dye that interacts specifically with dsDNA. Sample fluorescence was detected using a microplate reader (Wallac 1420 VICTOR F, Perkin Elmer, Shelton, CT, USA, or SpectraMax M3, Molecular Devices, San Jose, CA, USA) with a 485 nm emission filter. DNA from 12-month-old C57BL6 WT control mice was pooled and used as reference DNA after being diluted to 3 ng/μL as previously described [28].

### 2.3. PCR-Based Assays to Determine mtDNA Abundance and mtDNA Damage in Mouse Liver Tissue After AOM Treatment

To determine the relative abundance of mtDNA, we performed real-time PCR and evaluated the data using the 2^−ΔΔCT^ method described previously [31,32]. The nucleotide sequences for the PCR primers (Integrated DNA Technologies) were 5’-TCG GAG CCC ATA TAG CA-3’ (forward) and 5’-TTT CCG GCT AGA GGT GGG TA-3’ (reverse). These primers generated a 144 bp amplicon encompassing an mtDNA region from position 5587 to 5731. The nucleotide sequences of the primers for an nDNA amplicon were 5’-TTT GCT GTC CCA GTA GAG TAA G-3’ (forward) and 5’-CTC ACT AGA GTT GGT GGG AAT AG-3’ (reverse). The primers generated a 102 bp amplicon that encompassed a region of the hypoxanthine phosphoribosyl transferase (HPRT) gene. To determine the amplification efficiency of our primers, a standard curve was performed. The primer efficiency was determined to be 92.21% and 94.64% for mtDNA and nDNA, respectively. PCR reactions (10 ng of DNA) were prepared using iQ™ SYBR^®^ Green Supermix (Bio-Rad Laboratories, Hercules, CA, USA) following the manufacturer’s instructions. Briefly, the PCR reactions were performed with an initial denaturation for 10 min at 95 °C, 24 cycles of denaturation for 15 s at 95 °C, annealing/extension at 62 °C for 15 min, and a final melt curve of a single cycle with a denaturation for 15 s at 95 °C and annealing/extension at 62 °C for 1 min. Real-time PCR data were evaluated using QuantStudio^TM^ Real-Time PCR Software v1.3 (Thermo Fisher, Waltham, MA, USA).

DNA samples (3 ng) were diluted to obtain 1 ng of DNA/sample and tested in triplicate following iQ™ SYBR^®^ Green Supermix protocol in a 20 μL reaction. Both a No-DNA sample (in duplicate) and a reference DNA/calibrator in triplicate (from pooled DNA from the 6-month-old C57BL6 WT control mice) were included in the reaction. The assay was performed using mtDNA and nuclear DNA (nDNA) PCR primers separately. The mouse mtDNA (144 bp) and mouse nDNA (102 bp) fragments were amplified using an initial denaturation for 10 min at 95 °C, followed by 24 cycles (mtDNA amplicon) and 32 cycles (nDNA amplicon) of denaturation for 15 s at 95 °C, and annealing/extension at 62 °C and 55 °C for 15 min, respectively. Data were evaluated using the ΔΔCT (comparative CT) method using QuantStudio Software v1.3.

We performed qPCR assays to detect mtDNA damage in mouse liver tissue as previously described [33,34]. The primer nucleotide sequences for the amplification of the 10 kb mtDNA amplicon were: 5’-GCC AGC CTG ACC CAT AGC CAT AAT AT-3’ (forward) and 5’-GAG AGA TTT TAT GGG TGT AAT GCG G-3’ (reverse). The PCR amplification was performed using the Master Amp XL Polymerase (LGC Biosearch Technologies, Middleton, WI, USA) and Epicentre Premix E (LGC Biosearch Technologies, Middleton, WI, USA). The amplification profile for the 10 kb mtDNA fragment was: initial denaturation for 45 s at 94 °C, followed by 24 cycles and annealing/extension at 64 °C for 12 min, and a final extension at 72 °C for 10 min. The amplification data were expressed relative to the amplification of a reference DNA prepared from pooled DNA obtained from 6-month-old C57BL6 WT mice without AOM treatment. Lesions per 10 kilobases (Kb) pairs were calculated using the Poisson equation as previously described [33,34]. Briefly, the average lesion frequency per DNA strand can be calculated as λ = −ln A_D_/A_O_, where A_D_ is the amount of amplification of the damaged template and A_O_ represents the amount of amplification product from undamaged DNA. The results are expressed as a relative amplification ratio (A_D_/A_O_) and as lesion frequency per 10 Kb per DNA strand. The levels of mtDNA lesions in liver tissue from AOM-treated mice were calculated by comparing nontreated mice with treated mice from each strain separately. We normalized the amplification of the 10 kb mtDNA fragment with the data obtained using Real-Time PCR to determine relative mtDNA abundance.

### 2.4. Western Blot Analysis

For protein extraction, about 35 mg of liver tissue was minced to a fine powder using a mortar and pestle while keeping cryogenic conditions with liquid nitrogen. The minced liver tissue was transferred to a Potter-Evelhjem homogenizer tube and homogenized using 300 μL ice-cold Lysis Buffer (1% NP-40, Aprotinin (10 µg/mL), Antipain dihydrochloride (2 µg/mL), Benzamidine hydrochloride (5 mM), DL-Dithiothreitol (1 mM), Ethylenediaminetetraacetic Acid (1 mM), Ethylene glycol-bis [β-aminoethyl ether]-N,N, NʹNʹ-tetra acetic acid (1 mM), Leupeptin hydrochloride (10 µg/mL), Sodium Chloride (150 mM), Sodium Fluoride (5 mM), Sodium orthovanadate (1 mM), Phenylmethanesulfonyl fluoride (1 mM), Tris HCL (20 mM), Trypsin inhibitor II-O (10 µg/mL). Following homogenization, the sample was transferred to a microcentrifuge tube, incubated for 45 min at 4 °C, and centrifuged for 90 min at 14,000× *g* rpm at 4 °C. The supernatant containing the whole protein lysate was collected, and proteins were quantified and kept at −30 °C until analyzed.

Protein quantification (5 μL/sample) was performed using the colorimetric DC™ protein assay and the microplate reader SpectraMax M3. Twenty micrograms (20 μg) of protein were resolved by sodium dodecyl sulfate (SDS) polyacrylamide gel (12% separating gel and 4% stacking gel) electrophoresis for 60 min at 200 V. The proteins were transferred to a 0.2 µm Immuno-Blot polyvinylidene difluoride (PVDF) membrane, using the tank transfer method overnight at 12 V, 4 °C.

Membranes were blocked in 5% Bovine Serum Albumin (BSA) for 1 hr at room temperature, followed by overnight incubation with the primary antibodies ATP5β (Abcam #ab14730; 1:30,000; Waltham, MA, USA), β-Actin (Antibodies-online #ABIN1742508; 1:5000; Limerick, PA, USA), and GAPDH (Abcam #ab8245; 1:1000; Waltham, MA, USA) at 4 °C. Membranes were incubated with secondary antibodies IRDye^®^ 800CW Goat anti-Rabbit IgG (LICORbio # 926-32211; 1:25,000; Lincon, NE, USA) and IRDye^®^ 680RD Goat anti-Mouse IgG (LICORbio # 926-68070; 1:25,000; Lincon, NE, USA) for 1 h at room temperature, protected from light. The Odyssey^®^ DLx Imaging System (LICORbio, Lincon, NE, USA) was used to visualize the digital image of protein bands in the membranes. The intensity of the protein bands was quantified by the Image Studio™ Lite Software Version 5.2 (LICORbio, Lincon, NE, USA).

### 2.5. Histopathological Analysis

Livers from control and AOM-treated animals were fixed in 10% formaldehyde, embedded in paraffin, and stained using standard procedures. Specifically, paraffin blocks were sectioned at a thickness of 5 μm and stained with hematoxylin and eosin (H&E) to evaluate nuclear inclusions, portal inflammation, and ductular reaction, and Masson trichrome stain for fibrosis analysis. Slides with 3 liver sections per mouse were analyzed. Histological analyses were performed blindly by a pathologist.

Nuclear inclusions were defined as round intranuclear forms with sharp borders, a diameter > 1/3 that of the nucleus (to distinguish them from nucleoli), and the same tinctorial quality as the adjacent cytoplasm. Five fields at 200× magnification were scanned, and the average number of nuclear inclusions per field was reported.

Bile ductular proliferation, portal inflammation, and fibrosis were evaluated at 100× magnification. Bile ductular proliferation was scored using the following scale: none = 0; moderate ductular proliferation, not extending to the adjacent portal tract = 1; severe ductular proliferation, extending to the adjacent portal tract = 2; severe ductular proliferation, extending to the adjacent portal tract, with nodularity of the hepatic parenchyma = 3.

Portal inflammation was scored semi-quantitatively by evaluating lymphocyte and plasma cell infiltration as follows: none = 0; minimal inflammation confined to the portal areas = 1; mild inflammation extending into the parenchyma = 2.

Fibrosis scoring, which is a measure of the extent of chronic liver damage, was performed using the Batts-Ludwig scoring system [35]. Briefly, none = 0; portal expansion of scar tissue = 1; extension of scar into the periportal region with rare bridges of scar tissue = 2; bridging of scar tissue from portal tract to central vein = 3; cirrhosis (fibrous bands encircling nodular parenchyma) = 4.

Statistical analyses were performed for the following groups: Wild type (WT) Not Treated (NT) vs. WT AOM; WT NT vs. *Apex1^+/−^* AOM; and WT AOM vs. *Apex1^+/−^* AOM. No statistical analysis was performed using the *Apex1^+/−^* NT because the tissue was available from a single animal. Slices from this animal show scores that are very similar to those of WT NT animals.

### 2.6. Statistical Analysis

Data are expressed as mean ± the standard error (SEM). The Student’s *t*-test was used to determine the statistical difference between AOM-treated and untreated mice in wild-type and *Apex1^+/−^* mice. GraphPad PRISM version 10 was used for the statistical analyses.

## 3. Results

### 3.1. The Alkylating Agent AOM Causes a Decrease in mtDNA Abundance and an Increase in mtDNA Lesions in Mouse Liver Tissue

**Figure 1:**
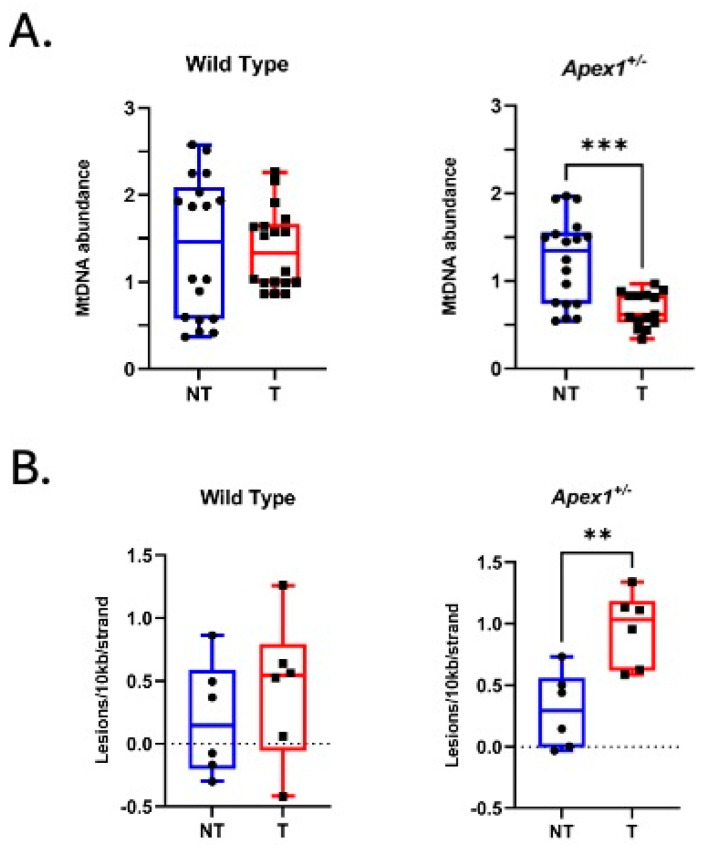
Mitochondrial DNA (mtDNA) abundance and mtDNA lesions in AOM-treated mice. DNA was extracted from the liver tissue of mice chronically treated with AOM and analyzed using quantitative PCR. (**A**) Relative abundance of mtDNA in WT and *Apex1^+/−^* mice. N = 6 animals per group. (**B**) Frequency of mtDNA lesions in nontreated (NT) and treated (T) WT and *Apex1^+/−^* mice. N = 6 mice per group. All PCR reactions were performed in triplicate. ** *p* = 0.0037, *** *p* = 0.0003 (*t*-test) NT vs. T mice. Data are presented as the mean ± SEM.

Previously, we demonstrated that the alkylating agent AOM induces mtDNA lesions and decreases the abundance of mtDNA in mouse colonocytes and that these effects are exacerbated in a mouse strain with a null allele of the *Apex1* gene [28]. Since AOM is metabolized and activated in the liver, we hypothesized that mtDNA lesions would be greater in *Apex1^+/−^* mice than in WT mice due to deficient DNA repair. We extracted DNA from liver tissue from the WT strain and *Apex1^+/−^* mice chronically treated with AOM. To compare the relative mtDNA abundance after AOM treatment, we used real-time PCR to determine the relative amplification of an mtDNA amp licon with respect to a nuclear amplicon and analyzed the data using the 2^−ΔΔCT^ method [31]. The results of these experiments show a 55% significant decrease (*p* = 0.0003) in mtDNA abundance in *Apex1^+/−^* mice with AOM treatment, which is not observed in WT mice (Figure 1A).

To detect mtDNA lesions, we used a PCR-based assay that can detect a variety of DNA lesions, such as base modifications, abasic sites, and single and double-strand breaks, to measure mtDNA damage [33,34,36]. In terms of mtDNA damage, a significant 3.2-fold increase in mice (*p* = 0.0037) lesions was observed in the *Apex1^+/−^* AOM-treated (from 0.30 lesions/10 kb/strand to 0.96 lesions/10 kb/strand) (Figure 1B). No significant differences were observed in the frequency of mtDNA lesions in WT AOM-treated mice (0.44 lesions/10 kb/strand) compared to WT Not-Treated mice (0.20 lesions/10 kb/strand) (*p* = 0.4335). Therefore, the liver from Apex1^+/−^ mice chronically treated with AOM shows a significant increase in mtDNA lesions and a concomitant decrease in mtDNA abundance.

### 3.2. Alkylation Damage Increases the β-F1-ATPase/GAPDH Ratio in Liver Tissue

**Figure 2:**
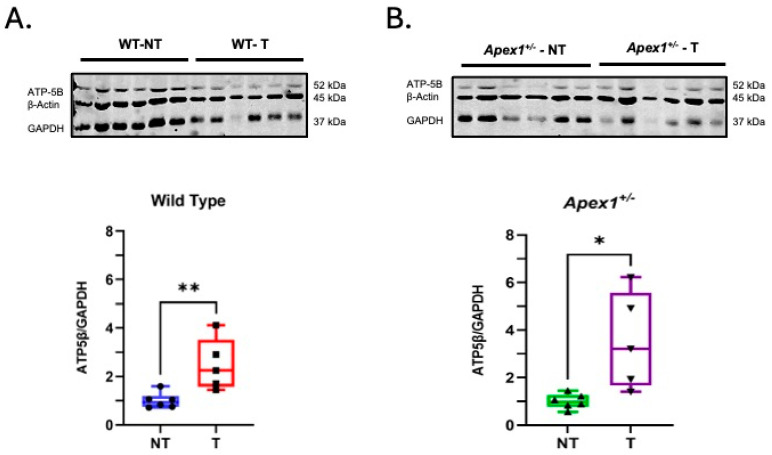
The alkylating agent AOM provokes an increase in the ATP5-β/GAPDH ratio in WT and *Apex1^+/−^* mice. (**A**) Representative Western Blots showing the expression of ATP5-β, β-Actin, and GAPDH are shown in WT and *Apex1^+/−^* AOM-treated mice (Upper panel). (**B**) Histograms represent the ATP5-β/GAPDH ratio of non-treated (NT) and treated (T) WT mice (left panel) and *Apex1^+/−^* mice (right panel). NT versus T WT mice (* *p* = 0.0134); NT versus T *Apex1^+/−^*mice ** *p* = 0.0098. N = 4–6 mice per group (non-treated and treated). Data are presented as the mean ± SEM. Western blot original images can be found in Figure S1.

Relative protein expression levels of β-F1-ATPase (involved in oxidative phosphorylation) and GAPDH (associated with glycolysis) have been used as a proxy of the cell bioenergetics signature [37,38]. Thus, the bioenergetics signature represents the relative contribution of oxidative phosphorylation and glycolysis in energy metabolism. Studies in hepatocellular carcinomas and colon cancer have shown that the β-F1-ATPase/GAPDH ratio is low in cancer cells [38]. To determine the effect of AOM-induced DNA damage on the β-F1-ATPase/GAPDH ratio in the liver, we performed a Western Blot analysis of protein samples isolated from liver tissue from mice chronically treated with AOM. Our results show a significant 1.5-fold (*p* = 0.0098) increase and a 2.5-fold (*p* = 0.0134) increase in the β-F1-ATPase/GAPDH ratio in WT mice and Apex1^+/−^ mice, respectively, after AOM treatment (Figure 2; original image can be found in Figure S1). These results indicate that AOM causes an increase in the β-F1-ATPase/GAPDH ratio in both mouse strains, with the increase being more pronounced in *Apex1^+/−^* mice.

### 3.3. Liver Histological Changes Induced by AOM

**Figure 3:**
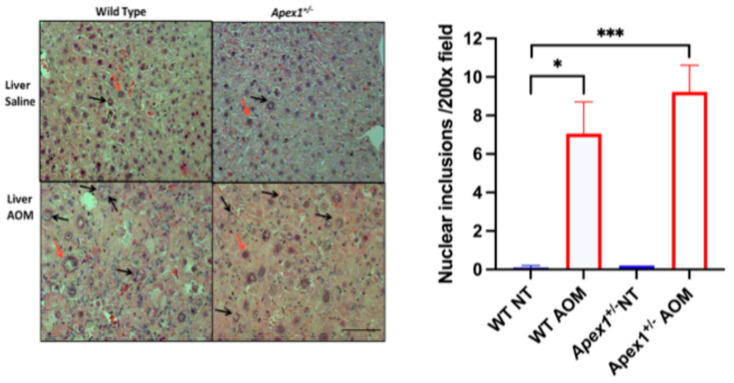
Nuclear inclusions induced by AOM in WT and *Apex1^+/−^*mice. H&E histological analysis for nuclear inclusions was performed in mice liver from 6-month-old C57BL/6 WT and *Apex1^+/−^*mice treated with a single injection of AOM (10 mg/kg) and sacrificed 6 months after the first injection. Red arrows show nuclei without inclusions. Black arrows show nuclei with nuclear inclusions. Nuclear inclusions were quantified in 5 fields at a magnification of 200×. Values represented mean ± SEM. N = 4 WT saline-treated group (NT), N = 7 WT AOM-treated, N = 1 *Apex1^+/−^* saline-treated group (NT), N = 7 *Apex1^+/−^*AOM-treated. The Student’s *t*-test was used to compare between groups. The *Apex1^+/−^* saline-treated group was excluded from the statistical analysis. Pictures are at 200× magnification. Bar size = 200 μm. * *p* < 0.05 and *** *p* < 0.001.

To assess the long-term effects of AOM treatment on histopathological liver lesions, we analyzed liver tissue for several markers of injury, including nuclear inclusions, bile ductular proliferation, portal inflammation, and fibrosis. Nuclear inclusions are invaginations of the cytoplasm into the nucleus that may contain degenerative cell organelles or glycogen deposits [39]. They have been observed during aging, in neoplastic hepatocytes, and virus-infected cells [40]. The average number of nuclear inclusions per 200× field was 0.1, 7.1, 0.2, and 9.2 for WT, WT AOM-treated, *Apex1^+/−^*, and *Apex1^+/−^* AOM-treated mice, respectively (Figure 3). Statistically significant differences were observed between WT vs. WT AOM-treated (*p* = 0.0125) and between WT vs. *Apex1^+/−^* AOM-treated (*p* = 0.0008) mice. There was no statistically significant difference in nuclear inclusions between WT AOM-treated and *Apex1^+/−^* AOM-treated mice (*p* = 0.3317).

**Figure 4:**
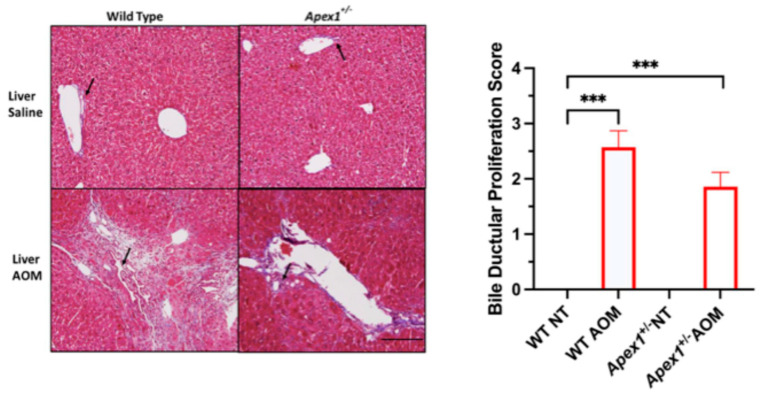
Ductular proliferation induced by AOM in WT and *Apex1^+/−^* mice. Histological analysis with Masson trichrome staining for ductular proliferation was performed in the liver of mice WT and *Apex1^+/−^* mice treated with a single injection of AOM (10 mg/kg) and sacrificed 6 months after the first injection. Scoring scale: 0 = none; 1 = moderate ductular proliferation, not extending to adjacent portal tract; 2 = severe ductular proliferation, extending to adjacent portal tract; 3 = severe ductular proliferation, extending to adjacent portal tract, with nodularity of hepatic parenchyma. Pictures at 200× magnification. The black arrows show regions of ductular proliferation. Values represented mean ± SEM. N = 4 WT saline-treated group (NT), N = 7 WT AOM-treated, N = 1 *Apex1^+/−^* saline-treated group (NT), N = 7 *Apex1^+/−^* AOM-treated. The Student’s *t*-test was used to compare between groups. The *Apex1^+/−^* saline-treated group was excluded from the statistical analysis. Bar size = 200 μm. *** *p* < 0.001.

Ductular reaction or proliferation refers to the appearance of biliary epithelial cells in the portal tracts of diseased livers and is associated with oval cell activation during liver regeneration [41]. Scores for bile ductular proliferation (semiquantitative, range 0–3) were 0.0, 2.6, 0.0, and 1.9 for WT, WT AOM-treated, *Apex1^+/−^*, and *Apex1^+/−^* AOM-treated mice, respectively (Figure 4). Statistically significant differences were observed between WT vs. WT AOM-treated (*p* = 0.0001) and WT vs. *Apex1^+/−^* AOM-treated mice (*p* = 0.0005). There was no statistically significant difference in bile ductular proliferation between WT AOM-treated and *Apex1^+/−^* AOM-treated mice (*p* = 0.0961).

**Figure 5:**
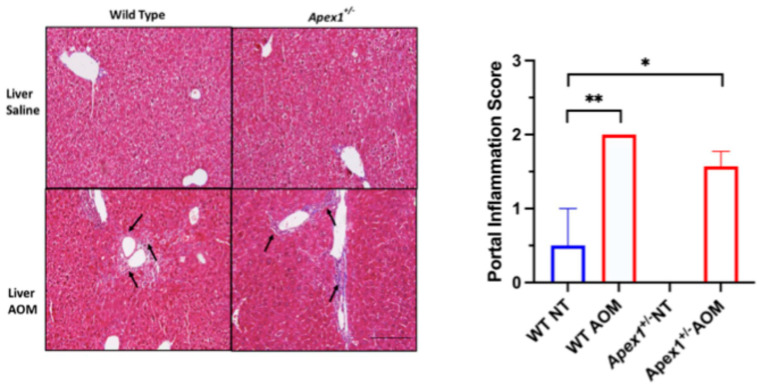
Portal inflammation induced by AOM in WT and *Apex1^+/−^*mice. Histological analysis with Masson trichrome staining for portal inflammation was performed on the liver of mice WT and *Apex1^+/−^* mice treated with a single injection of AOM (10 mg/kg) and sacrificed 6 months after the first injection. Portal inflammation was scored by evaluating lymphocyte and plasma cell infiltration as follows: 0 = none; 1 = minimal, inflammation confined to portal areas; 2 = mild, inflammation extending to the parenchyma. Pictures at 200× magnification. The black arrows show lymphocyte infiltration. Values represented mean ± SEM. N = 4 WT saline-treated group (NT), N = 7 WT AOM-treated, N = 1 *Apex1^+/−^* saline-treated group (NT), N = 7 *Apex1^+/−^*AOM-treated. Pictures were taken at 200× magnification. The Student’s *t*-test was used to compare between groups. The *Apex1^+/−^*saline-treated group was excluded from the statistical analysis. Bar size = 200 μm. * *p* < 0.05 and ** *p* < 0.001.

Portal inflammation is characterized by inflammatory infiltrates of lymphocytes, plasma cells, and occasionally eosinophils and monocytes [42]. The portal inflammation scores (semiquantitative, range 0–2) for the WT, WT AOM-treated, *Apex1^+/−^*, and *Apex1^+/−^* AOM-treated mice were the following: 0.5, 2.0, 0, and 1.6, respectively (Figure 5). Statistically significant differences were observed between WT vs. WT AOM-treated mice (*p* = 0.0025) and WT NT vs. *Apex1^+/−^* AOM-treated mice (*p* = 0.0425). We observed a 20% reduction in portal inflammation in *Apex1^+/−^* AOM-treated mice compared to WT AOM-treated mice; however, this decrease did not reach statistical significance, although it was borderline (*p* = 0.0554).

**Figure 6:**
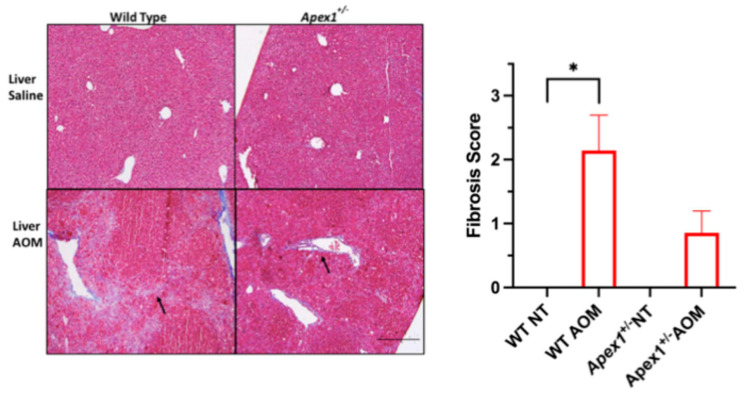
AOM-induced fibrosis in WT and *Apex1^+/−^*mice. Histological analysis with Masson trichrome staining for fibrosis was performed on the liver of WT and *Apex1^+/−^* mice that were treated with a single injection of AOM (10 mg/kg) and sacrificed 6 months after the first injection. Scoring scale: 0 = none; 1= portal expansion of scar tissue; 2 = rare bridges of scar tissue; 3 = bridging of scar tissue. For each parameter, a higher score indicates increased liver tissue damage. The black arrow is showing bridging. Pictures at 100× magnification. Black arrows show scarred tissue. Values represented mean ± SEM. N = 4 WT saline-treated group (NT), N = 7 WT AOM-treated, N = 1 *Apex1^+/−^* saline-treated group (NT), N = 7 *Apex1^+/−^* AOM-treated. The Student’s *t*-test was used to compare between groups. The *Apex1^+/−^* saline-treated group was excluded from the statistical analysis. Bar size = 200 μm. * *p* < 0.05.

Liver fibrosis consists of the accumulation of extracellular matrix proteins, including collagen. It may lead to other chronic liver diseases such as cirrhosis and cancer [43]. Fibrosis scores using the Batts-Ludwig system (range 0–4) were 0.0, 2.1, 0.0, and 0.9 for WT, WT AOM-treated, *Apex1^+/−^*, and *Apex1^+/−^* AOM-treated mice, respectively (Figure 6). Statistical analysis shows a significant difference between WT vs. WT AOM-treated mice (*p* = 0.0188). However, contrary to the histopathological parameters mentioned above, there is no significant increase in the score of the WT NT and *Apex1^+/−^* AOM-treated mice (*p* = 0.0956). The *p*-value for the comparison between WT AOM-treated and *Apex1^+/−^* AOM-treated mice was not statistically significant at the 0.05 threshold (*p* = 0.0711).

Taken together, these findings indicate that AOM treatment led to marked histopathological alterations in the liver of WT mice, including nuclear inclusions, bile ductular proliferation, portal inflammation, and fibrosis. *Apex1^+/−^* AOM-treated mice exhibited similar increases, except for fibrosis. Although no significant differences were detected between WT AOM-treated and *Apex1^+/−^* AOM-treated mice across most parameters, the reduced fibrotic response in *Apex1^+/−^* mice suggests that decreased APE1 selectively attenuates AOM-induced fibrogenesis.

## 4. Discussion

In this work, we explored the contribution of APE1 in maintaining mtDNA integrity after long-term exposure to AOM in the liver of mice haploinsufficient for *Apex1*. We reported a significant increase in mtDNA damage and a decreased abundance of mtDNA in liver tissue from *Apex1^+/−^* mice treated with AOM. We also detected a stronger increase in the β-F1-ATPase/GAPDH ratio in *Apex1^+/−^* mice compared to WT mice. All these changes occur in the context of notable histological alterations, such as increased nuclear inclusions and ductular proliferation in AOM-induced liver tissue in both mouse strains, while fibrosis was attenuated in the *Apex1^+/−^* mice. These results show that APE1 is essential for preserving mitochondrial DNA integrity and modulating the pathological response.

Wild-type mice treated with AOM do not show a significant increase in liver mtDNA lesions, while a significant increase was observed in the *Apex1^+/−^* AOM-treated mice. Concomitant to mtDNA damage, we also observe a significant decrease in relative mtDNA abundance in *Apex1^+/−^* AOM-treated mice, but not in WT AOM-treated mice. We interpret these results as evidence of deficient mitochondrial DNA (mtDNA) repair in the liver of *Apex1^+/−^*. Alternatively (or simultaneously), decreased REDOX activity in *Apex1^+/−^* mice may result in increased ROS generation, contributing to mtDNA lesion burden. On the other hand, mild chronic ROS levels may stimulate mitochondrial quality mechanisms, such as mitophagy, which may maintain a population of healthy mitochondria, accounting for the enhanced bioenergetics signature in these mice. Our previous work in colonocytes from mice treated with AOM also shows that AOM treatment leads to loss of mtDNA abundance in both WT and *Apex1^+/−^* mice [28]. Our studies underscore the role of APE1 in maintaining mtDNA integrity after AOM-induced injury, not only in colorectal tissue but also in the liver. It is noteworthy to discuss the nature of the mtDNA lesions detected in our study. The liver samples analyzed were taken 5 months after the last AOM injection. By that time, all the original alkylating DNA lesions should have been repaired. Thus, there must be another source of mtDNA damage. Given that our histopathological analyses show an increase in inflammation markers, we propose that these mtDNA lesions are due to long-term oxidative stress. Since the PCR-based approach we utilized to detect DNA damage does not discriminate between different DNA lesions that can block the movement of the polymerase during PCR, we cannot specifically name the type of lesions predominant in the samples. Thus, further studies are needed to characterize the nature of the mtDNA lesions and their source after long-term exposure to AOM.

Studies on hepatocellular carcinomas have revealed significant reductions in the protein levels of mitochondrial components, such as β-F1-ATPase, providing cancer cells with a bioenergetics phenotype characterized by enhanced glycolysis and concomitant downregulation of OXPHOS [38,44,45]. However, our study shows an increase in the β-F1-ATPase/GAPDH ratio in both WT and *Apex1^+/−^* mice treated with AOM (Figure 2). The difference in the direction of the response to AOM in our study may represent the status of the non-cancerous state of the liver tissue used in our study versus the cancerous state of the tissues in the studies referenced above. In addition to inducing apoptosis, cell cycle arrest, and DNA damage response, the p53 protein also regulates metabolism by repressing the GLUT1 and GLUT4 transporters [46,47]. Therefore, in preneoplastic tissue, where p53 mutations have not occurred, the bioenergetics response may reflect an enhanced OXPHOS over glycolysis, such as the one we report in this work. Furthermore, recent work shows that the DNA damage response protein CHK2 regulates both glycolysis and oxidative metabolism; thus, in advanced stages of liver damage, the combination of the effects of p53 on the GLUT transporters, mentioned above, and increased energy demand could be reflected in the increase in the β-F1-ATPase/GAPDH ratio reported in our study [4].

Suganya et al. [48] reported suppression of OXPHOS in mouse embryonic fibroblasts (MEFs) with markedly reduced or absent APE1 expression. As mentioned above, our study demonstrates an enhanced bioenergetics capacity in liver tissues from AOM-treated *Apex1^+/–^* mice. This apparent discrepancy can be attributed to differences in (i) the degree of APE1 deficiency, (ii) cellular context, and (iii) experimental conditions. First, partial reduction in APE1 levels in *Apex1^+/–^* may preserve essential repair and redox functions that promote compensatory responses that transiently enhance OXPHOS. In contrast, the near-complete loss of APE1 in MEFs, as used by Suganya et al., results in a severe bioenergetics deficit, as evidenced by their inability to increase oxygen consumption when challenged with FCCP. Second, tissue-specific metabolic demands and the in vivo preneoplastic microenvironment following chronic AOM exposure differ profoundly from those of immortalized MEFs under basal culture conditions. Finally, the preneoplastic liver we analyzed probably retains intact tumor-suppressor signaling (e.g., p53, CHK2) and thus may favor an oxidative metabolic program, whereas MEFs transformed with SV40 T-antigen exhibit metabolic rewiring typical of transformed cells. Together, these factors explain why partial APE1 insufficiency can stimulate OXPHOS in vivo, whereas near-complete depletion suppresses it in vitro. We used the β-F1-ATPase/GAPDH ratio as a proxy indicator of mitochondrial bioenergetics due to the limited availability of frozen tissue, which precluded more comprehensive analyses of mitochondrial function. While the measurement of these protein levels provides insight into potential shifts in bioenergetic pathways, it does not directly assess mitochondrial respiratory capacity or efficiency. Future studies using freshly isolated tissues will provide the opportunity to validate and build upon these findings with more direct approaches, such as measuring oxygen consumption rate.

Long-term treatment with AOM resulted in significant histological changes in the liver of both WT and *Apex1^+/−^* mice, including increased nuclear inclusions, ductular proliferation, portal inflammation, and fibrosis. Nuclear inclusions were consistent with oxidative-stress-induced alterations previously described in rodents [19,49,50]. Ductular proliferation reflects regenerative and potentially preneoplastic processes [51]. Portal inflammation was also evident, and although *Apex1^+/−^* AOM-treated mice displayed a modest reduction compared to WT AOM-treated mice, this difference did not reach statistical significance. Fibrosis, however, was more pronounced in WT AOM-treated mice, whereas *Apex1^+/−^* AOM-treated mice showed an attenuated response. Together, these findings confirm that AOM provokes broad histopathological injury, with APE1 haploinsufficiency selectively influencing inflammatory and fibrotic outcomes.

Our findings underscore the delicate balance maintained by APE1, where its insufficiency can simultaneously compromise mtDNA genome integrity while mitigating inflammatory damage, highlighting its multifaceted role in cellular physiology and disease. Danger-associated molecular patterns (DAMPs) are molecules (such as mtDNA fragments) released by damaged or dying cells in response to various stressors, including genotoxic stress, which activate the cGAS-STING signaling cascade and induce the innate immune response [52]. Therefore, reduced APE1-mediated mtDNA repair, in response to chronic AOM treatment, should be expressed as enhanced inflammation and fibrosis in APE1 haploinsufficient animals compared to WT. However, our results suggest a blunted response. These observations lead us to propose that the cGAS-STING signaling cascade may be reduced in the livers of *Apex1^+/−^* mice. Support for this hypothesis comes from studies showing that APE1 endonuclease and redox activities are involved in innate immunity [21]. In addition, pharmacological inhibition of APE1’s redox function prevents the activation of NK-kB, IL-6, and IL-8 induced by THF-α and free fatty acids in the human hepatocellular carcinoma cell line JHH-6 [53]. This observation provides a mechanistic model of how APE1 haploinsufficiency can lead to reduced inflammatory response after genotoxic injury. The role of APE1 in modulating the cGAS-STING pathway may be tissue-specific, considering recent findings showing that in lung adenocarcinoma, APE1 contributes to radiation resistance by inhibiting this pathway [54]. Further studies are needed to identify which of the various biochemical functions of APE1 (and its tissue specificity) are most relevant for regulating the cGAS-STING signaling cascade.

While our findings highlight the role of APE1 in maintaining mtDNA integrity, it is important to recognize that APE1 also exerts critical nuclear and redox regulatory functions. Therefore, the phenotypic changes observed following AOM exposure likely reflect the combined effects of APE1 deficiency in both compartments rather than mitochondrial mechanisms alone. Future studies employing compartment-specific APE1 mutants will be necessary to dissect the relative contribution of mitochondrial versus nuclear APE1 functions to the long-term hepatic response to AOM-induced genotoxic stress.

## 5. Conclusions

In summary, APE haploinsufficiency compromises mitochondrial genome integrity, leading to increased mtDNA damage and altered bioenergetic and histopathological responses to AOM. Interestingly, despite these vulnerabilities, *Apex1^+/−^* mice exhibited attenuated fibrosis, underscoring APE1’s dual role in mitochondrial stability and immune regulation. These findings point to mtDNA integrity as a central factor linking genotoxic stress to chronic liver injury and warrant further mechanistic investigation.

## Data Availability

The datasets used during the current study are available from the corresponding author on request.

## Supplementary Materials

The following supporting information can be downloaded at: https://www.mdpi.com/article/10.3390/biom15121706/s1, Figure S1: Western Blot (uncropped/unedited image) showing the expression of ATP5-β, β-Actin, and GAPDH in wild type (WT) and Apex1^+/−^ mice after AOM treatment.

## Abbreviations

The following abbreviations are used in this manuscript:

APE1	Apurinic/apyrimidinic endonuclease 1
*Apex1*	Mouse gene encoding APE1
AOM	Azoxymethane
BER	Base Excision Repair
CRC	Colorectal Cancer
HCC	Hepatocellular Carcinoma
ROS	Reactive Oxygen Species
mtDNA	Mitochondrial DNA
nDNA	Nuclear DNA
AP sites	Apurinic/apyrimidinic sites
MAM	Methylazoxymethanol
PCR	Polymerase Chain Reaction
qPCR	Quantitative PCR
EtBr	Ethidium Bromide
HPRT	Hypoxanthine Guanine Phosphoribosyltransferase
dsDNA	Double-stranded DNA
SDS	Sodium Dodecyl Sulfate
PVDF	Polyvinylidene Difluoride
BSA	Bovine Serum Albumin
IgG	Immunoglobulin G
SEM	Standard Error of the Mean
WT	Wild-Type
NT	Non-treated
OXPHOS	Oxidative Phosphorylation
GLUT1/GLUT4	Glucose Transporters 1 and 4
CHK2	Checkpoint Kinase 2
cGAS-STING	Cyclic GMP-AMP Synthase–Stimulator of Interferon Genes
DAMPs	Danger-Associated Molecular Patterns
IL-6, IL-8	Interleukins 6 and 8
NF-κB	Nuclear Factor kappa-light-chain-enhancer of activated B cells
THF-α	Tumor Necrosis Factor-alpha
AVMA	American Veterinary Medical Association

## References

[1] Auger C., Alhasawi A., Contavadoo M., Appanna V.D. (2015). Dysfunctional mitochondrial bioenergetics and the pathogenesis of hepatic disorders. Front. Cell Dev. Biol..

[2] Balogh J., Victor D., Asham E.H., Burroughs S.G., Boktour M., Saharia A., Li X., Ghobrial R.M., Monsour H.P. (2016). Hepatocellular carcinoma: A review. J. Hepatocell. Carcinoma.

[3] Loureiro D., Tout I., Narguet S., Bed C.M., Roinard M., Sleiman A., Boyer N., Pons-Kerjean N., Castelnau C., Giuly N. (2023). Mitochondrial stress in advanced fibrosis and cirrhosis associated with chronic hepatitis B, chronic hepatitis C, or nonalcoholic steatohepatitis. Hepatology.

[4] Lulli M., Del Coco L., Mello T., Sukowati C., Madiai S., Gragnani L., Forte P., Fanizzi F.P., Mazzocca A., Rombouts K. (2021). DNA Damage Response Protein CHK2 Regulates Metabolism in Liver Cancer. Cancer Res..

[5] Sun Z., Zhu Y., Aminbuhe, Fan Q., Peng J., Zhang N. (2018). Differential expression of APE1 in hepatocellular carcinoma and the effects on proliferation and apoptosis of cancer cells. Biosci. Trends.

[6] Xuan W., Song D., Yan Y., Yang M., Sun Y. (2020). A Potential Role for Mitochondrial DNA in the Activation of Oxidative Stress and Inflammation in Liver Disease. Oxid. Med. Cell. Longev..

[7] Zaidieh T., Smith J.R., Ball K.E., An Q. (2021). Mitochondrial DNA abnormalities provide mechanistic insight and predict reactive oxygen species-stimulating drug efficacy. BMC Cancer.

[8] Yamada S., Nomoto S., Fujii T., Kaneko T., Takeda S., Inoue S., Kanazumi N., Nakao A. (2006). Correlation between copy number of mitochondrial DNA and clinico-pathologic parameters of hepatocellular carcinoma. Eur. J. Surg. Oncol..

[9] Yin P.H., Lee H.C., Chau G.Y., Wu Y.T., Li S.H., Lui W.Y., Wei Y.H., Liu T.Y., Chi C.W. (2004). Alteration of the copy number and deletion of mitochondrial DNA in human hepatocellular carcinoma. Br. J. Cancer.

[10] Yu C., Wang X., Huang L., Tong Y., Chen L., Wu H., Xia Q., Kong X. (2018). Deciphering the Spectrum of Mitochondrial DNA Mutations in Hepatocellular Carcinoma Using High-Throughput Sequencing. Gene Expr..

[11] Hsu C.-C., Lee H.-C., Wei Y.-H. (2013). Mitochondrial DNA alterations and mitochondrial dysfunction in the progression of hepatocellular carcinoma. World J. Gastroenterol..

[12] Bissahoyo A., Pearsall R.S., Hanlon K., Amann V., Hicks D., Godfrey V.L., Threadgill D.W. (2005). Azoxymethane Is a Genetic Background-Dependent Colorectal Tumor Initiator and Promoter in Mice: Effects of Dose, Route, and Diet. Toxicol. Sci..

[13] Chen J., Huang X.F. (2009). The signal pathways in azoxymethane-induced colon cancer and preventive implications. Cancer Biol. Ther..

[14] Matkowskyj K.A., Marrero J.A., Carroll R.E., Danilkovich A.V., Green R.M., Benya R.V. (1999). Azoxymethane-induced fulminant hepatic failure in C57BL/6J mice: Characterization of a new animal model. Am. J. Physiol..

[15] Rosenberg D.W., Giardina C., Tanaka T. (2009). Mouse models for the study of colon carcinogenesis. Carcinogenesis.

[16] Beranek D.T. (1990). Distribution of methyl and ethyl adducts following alkylation with monofunctional alkylating agents. Mutat. Res..

[17] Fahrer J., Christmann M. (2023). DNA Alkylation Damage by Nitrosamines and Relevant DNA Repair Pathways. Int. J. Mol. Sci..

[18] Rahman K.M.W., Sugie S., Tanaka T., Mori H., Reddy B.S. (2001). Effect of types and amount of dietary fat during the initiation phase of hepatocarcinogenesis. Nutr. Cancer.

[19] Waggie K.S., Corulli L.R., Cecil D., Rodmaker E.R., Walsh C., Disis M.L. (2022). Unexpected Liver and Kidney Pathology in C57BL/6J Mice Fed a High-fat Diet and Given Azoxymethane to Induce Colon Cancer. Comp. Med..

[20] Whitaker A.M., Freudenthal B.D. (2018). APE1: A skilled nucleic acid surgeon. DNA Repair..

[21] Oliveira T.T., Coutinho L.G., de Oliveira L.O.A., de Souza A.R., Farias G.C., Agnez-Lima L.F. (2022). APE1/Ref-1 Role in Inflammation and Immune Response. Front. Immunol..

[22] Mijit M., Kpenu E., Chowdhury N.N., Gampala S., Wireman R., Liu S., Babb O., Georgiadis M.M., Wan J., Fishel M.L. (2024). In vitro and In vivo evidence demonstrating chronic absence of Ref-1 Cysteine 65 impacts Ref-1 folding configuration, redox signaling, proliferation and metastasis in pancreatic cancer. Redox. Biol..

[23] Caston A.R., Gampala S., Armstrong L., Messmann R.A., Fishel M.L., Kelley M.R. (2021). The multifunctional APE1 DNA repair-redox signaling protein as a drug target in human disease. Drug Discov. Today.

[24] Gampala S., Moon H.R., Wireman R., Peil J., Kiran S., Mitchell D.K., Brewster K., Mang H., Masters A., Bach C. (2024). New Ref-1/APE1 targeted inhibitors demonstrating improved potency for clinical applications in multiple cancer types. Pharmacol. Res..

[25] Sahakian L., Robinson A.M., Sahakian L., Stavely R., Kelley M.R., Nurgali K. (2023). APE1/Ref-1 as a Therapeutic Target for Inflammatory Bowel Disease. Biomolecules.

[26] Chattopadhyay R., Wiederhold L., Szczesny B., Boldogh I., Hazra T.K., Izumi T., Mitra S. (2006). Identification and characterization of mitochondrial abasic (AP)-endonuclease in mammalian cells. Nucleic Acids Res..

[27] Mitra S., Izumi T., Boldogh I., Bhakat K.K., Chattopadhyay R., Szczesny B. (2007). Intracellular trafficking and regulation of mammalian AP-endonuclease 1 (APE1), an essential DNA repair protein. DNA Repair..

[28] Ballista-Hernández J., Martínez-Ferrer M., Vélez R., Climent C., Sánchez-Vázquez M.M., Torres C., Rodríguez-Muñoz A., Ayala-Peña S., Torres-Ramos C.A. (2017). Mitochondrial DNA integrity is maintained by APE1 in carcinogen-induced colorectal cancer. Mol. Cancer Res..

[29] Ludwig D.L., MacInnes M.A., Takiguchi Y., Purtymun P.E., Henrie M., Flannery M., Meneses J., Pedersen R.A., Chen D.J. (1998). A murine AP-endonuclease gene-targeted deficiency with post-implantation embryonic progression and ionizing radiation sensitivity. Mutat. Res..

[30] Huamani J., McMahan C.A., Herbert D.C., Reddick R., McCarrey J.R., MacInnes M.I., Chen D.J., Walter C.A. (2004). Spontaneous Mutagenesis Is Enhanced in Apex Heterozygous Mice. Mol. Cell. Biol..

[31] Rao X., Lai D., Huang X. (2013). A new method for quantitative real-time polymerase chain reaction data analysis. J. Comput. Biol..

[32] Gonzalez-Hunt C.P., Rooney J.P., Ryde I.T., Anbalagan C., Joglekar R., Meyer J.N. (2016). PCR-Based Analysis of Mitochondrial DNA Copy Number, Mitochondrial DNA Damage, and Nuclear DNA Damage. Curr. Protoc. Toxicol..

[33] Ayala-Torres S., Chen Y., Svoboda T., Rosenblatt J., Van Houten B. (2000). Analysis of gene-specific DNA damage and repair using quantitative polymerase chain reaction. Methods.

[34] Furda A., Santos J.H., Meyer J.N., Van Houten B. (2014). Quantitative PCR-based measurement of nuclear and mitochondrial DNA damage and repair in mammalian cells. Methods Mol. Biol..

[35] Goodman Z.D. (2007). Grading and staging systems for inflammation and fibrosis in chronic liver diseases. J. Hepatol..

[36] Santos J.H., Meyer J.N., Mandavilli B.S., Van Houten B. (2006). Quantitative PCR-based measurement of nuclear and mitochondrial DNA damage and repair in mammalian cells. Methods Mol. Biol..

[37] Cuezva J.M., Krajewska M., de Heredia M.L., Krajewski S., Santamaría G., Kim H., Zapata J.M., Marusawa H., Chamorro M., Reed J.C. (2002). The bioenergetic signature of cancer: A marker of tumor progression. Cancer Res..

[38] Cuezva J.M., Ortega Á.D., Willers I., Sánchez-Cenizo L., Aldea M., Sánchez-Aragó M. (2009). The tumor suppressor function of mitochondria: Translation into the clinics. Biochim. Biophys. Acta.

[39] Schwertheim S., Kälsch J., Jastrow H., Schaefer C.M., Theurer S., Ting S., Canbay A., Wedemeyer H., Schmid K.W., Baba H.A. (2020). Characterization of two types of intranuclear hepatocellular inclusions in NAFLD. Sci. Rep..

[40] Thoolen B., Maronpot R.R., Harada T., Nyska A., Rousseaux C., Nolte T., Malarkey D.E., Kaufmann W., Küttler K., Deschl U. (2010). Proliferative and nonproliferative lesions of the rat and mouse hepatobiliary system. Toxicol. Pathol..

[41] Chen Y.K., Zhao X.X., Li J.G., Lang S., Wang Y.M. (2006). Ductular proliferation in liver tissues with severe chronic hepatitis B: An immunohistochemical study. World J. Gastroenterol..

[42] Brunt E.M., Kleiner D.E., Wilson L.A., Unalp A., Behling C.E., Lavine J.E., Neuschwander-Tetri B.A. (2009). Portal chronic inflammation in nonalcoholic fatty liver disease (NAFLD): A histologic marker of advanced NAFLD-Clinicopathologic correlations from the nonalcoholic steatohepatitis clinical research network. Hepatology.

[43] Bataller R., Brenner D.A. (2005). Liver fibrosis. J. Clin. Invest..

[44] Lee H.C., Wei Y.H. (2009). Mitochondrial DNA instability and metabolic shift in human cancers. Int. J. Mol. Sci..

[45] Sanchez-Arago M., Chamorro M., Cuezva J.M. (2010). Selection of cancer cells with repressed mitochondria triggers colon cancer progression. Carcinogenesis.

[46] Liang Y., Liu J., Feng Z. (2013). The regulation of cellular metabolism by tumor suppressor p53. Cell Biosci..

[47] Schwartzenberg-Bar-Yoseph F., Armoni M., Karnieli E. (2004). The tumor suppressor p53 down-regulates glucose transporters GLUT1 and GLUT4 gene expression. Cancer Res..

[48] Suganya R., Chakraborty A., Miriyala S., Hazra T.K., Izumi T. (2015). Suppression of oxidative phosphorylation in mouse embryonic fibroblast cells deficient in apurinic/apyrimidinic endonuclease. DNA Repair..

[49] Waly M.I., Al Alawi A.A., Al Marhoobi I.M., Rahman M.S. (2016). Red Seaweed (Hypnea Bryodies and Melanothamnus Somalensis) Extracts Counteracting Azoxymethane-Induced Hepatotoxicity in Rats. Asian Pac. J. Cancer Prev..

[50] Ward J.M. (1975). Dose response to a single injection of azoxymethane in rats. Induction of tumors in the gastrointestinal tract, auditory sebaceous glands, kidney, liver and preputial gland. Vet. Pathol..

[51] Gouw A.S.H., Clouston A.D., Theise N.D. (2011). Ductular reactions in human liver: Diversity at the interface. Hepatology.

[52] Chen R., Du J., Zhu H., Ling Q. (2021). The role of cGAS-STING signalling in liver diseases. JHEP Rep..

[53] Cesaratto L., Codarin E., Vascotto C., Leonardi A., Kelley M.R., Tiribelli C., Tell G. (2013). Specific inhibition of the redox activity of ape1/ref-1 by e3330 blocks tnf-α-induced activation of IL-8 production in liver cancer cell lines. PLoS ONE.

[54] Zhou J., Wei Z., Yang C., Jia D., Pan B., Zeng Y., Sun D., Yu Y. (2023). APE1 promotes radiation resistance against radiation-induced pyroptosis by inhibiting the STING pathway in lung adenocarcinoma. Transl. Oncol..

