# Supplementary material for: Mitochondrial DNA Damage and Histological Features in Liver Tissue of Azoxymethane-Treated Apex1 Haploinsufficient Mice
Source: Biomolecules. 2025 Dec 6;15(12):1706. doi: 10.3390/biom15121706 (PMC12731096; doi:10.3390/biom15121706)
Supplement: Supplementary file 1 [file biomolecules-15-01706-s001.zip › biomolecules-3944305-supplementary.pdf]

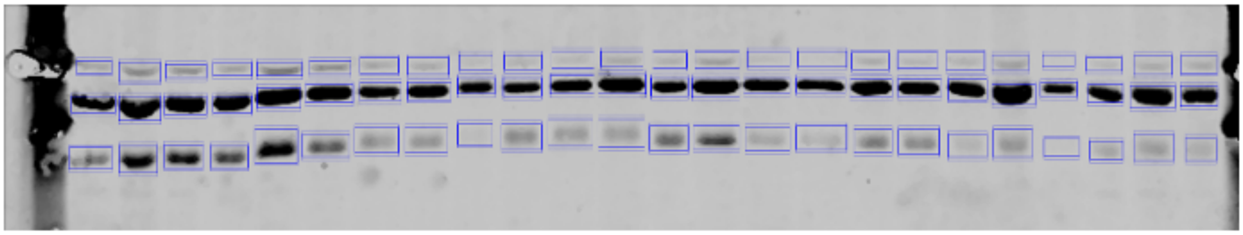

Supplementary Information Figure S1.

Western Blot (uncropped/unedited image) showing the expression of ATP5-β, β-Actin, and GAPDH in wild type (WT) and *Apex1*$^{+/-}$ mice after AOM treatment. M, protein size markers are in the first and last lanes (Precision Plus Kaleidoscope Standards, BIO-RAD Catalog #161-0375). The Odyssey® DLx Imaging System was used to obtain the digital image of protein bands in the membrane. In each lane, the order of the bands corresponds to ATP5β, β-Actin, and GAPDH, respectively. The intensity of the protein bands was quantified by Image Studio Lite Ver 5.2. Trim Signals shown below in the same order (ATP5-β, β-Actin, and GAPDH). For simplicity, Figure 2 in the manuscript presents the image in two sections: WT animals and *Apex1*$^{+/-}$ animals. In addition, the marker lanes (M) are not included. Instead, the expected protein size are indicated.

Image Name	Channel	Name	Area	Trim Signal
0017552_05	700	00001	147	170.3918586
0017552_05	700	00002	275	357.065567
0017552_05	700	00003	192	301.825431
0017552_05	700	00004	184	186.8230422
0017552_05	700	00005	216	597.2673788
0017552_05	700	00006	200	357.4674479
0017552_05	700	00007	207	178.4159947
0017552_05	700	00008	250	145.0562604
0017552_05	700	00009	180	62.82118056
0017552_05	700	00010	198	113.5427734
0017552_05	700	00011	250	78.7580372
0017552_05	700	00012	225	160.5281135
0017552_05	700	00013	180	111.71875
0017552_05	700	00014	234	251.5978589
0017552_05	700	00015	225	55.903633
0017552_05	700	00016	261	40.18037733
0017552_05	700	00017	220	126.6775174
0017552_05	700	00018	216	105.9358259
0017552_05	700	00019	230	108.9873798
0017552_05	700	00020	220	221.0047743
0017552_05	700	00021	133	13.56685014
0017552_05	700	00022	168	63.96278783
0017552_05	700	00023	253	156.345878
0017552_05	700	00024	220	152.4348958
0017552_05	700	00025	325	818.8747367
0017552_05	700	00026	368	2382.121517
0017552_05	700	00027	322	2007.258392
0017552_05	700	00028	299	1292.134387
0017552_05	700	00029	500	3186.703559
0017552_05	700	00030	392	1378.053628
0017552_05	700	00031	390	581.1199951
0017552_05	700	00032	375	566.8783272
0017552_05	700	00033	252	66.30931332
0017552_05	700	00034	360	726.1892361
0017552_05	700	00035	416	542.1795213
0017552_05	700	00036	459	554.2876482
0017552_05	700	00037	345	925.0977662
0017552_05	700	00038	378	1550.742496
0017552_05	700	00039	364	371.7617902
0017552_05	700	00040	420	268.1271701
0017552_05	700	00041	322	742.4867188
0017552_05	700	00042	360	685.9722222
0017552_05	700	00043	322	167.3194235
0017552_05	700	00044	391	549.7215192
0017552_05	700	00045	210	32.58367599
0017552_05	700	00046	220	223.8107639
0017552_05	700	00047	384	489.6893064
0017552_05	700	00048	252	214.151213
0017552_05	800	00049	260	1933.033583
0017552_05	800	00050	375	2659.657706
0017552_05	800	00051	275	2961.676099
0017552_05	800	00052	230	2022.493497
0017552_05	800	00053	312	3894.320063
0017552_05	800	00054	308	3706.251988
0017552_05	800	00055	275	1105.922763
